# Editorial: Advancements in understanding and managing preeclampsia: exploring molecular mechanisms, biomarkers, and clinical implications

**DOI:** 10.3389/fcell.2025.1718459

**Published:** 2025-11-07

**Authors:** Rajni Kant, Rajesh Kumar Manne, Subhradip Karmakar

**Affiliations:** 1 Department of Pathology, Duke University Medical Center, Duke University School of Medicine, Durham, NC, United States; 2 Department of Biochemistry, All India Institute of Medical Sciences, New Delhi, India

**Keywords:** preeclampsia, biomarker, metabolic reprograming, placenta, mitochondria

## Introduction

Preeclampsia remains one of the most serious and complex challenges in obstetrics affecting 3%–8% of pregnancies worldwide and standing as a leading cause of maternal and perinatal morbidity and mortality. Despite decades of research, this multisystem disorder, characterized by new-onset hypertension and organ dysfunction after 20 weeks of gestation, remains incompletely understood and inadequately managed. However, recent advances in genomics, metabolomics, and systems biology are revolutionizing our understanding of preeclampsia’s molecular underpinnings, offering unprecedented opportunities for precision medicine approaches to prediction, prevention, and treatment. This Research Topic brings together complimentary studies that advance insights into molecular and cellular mechanisms while outlining translational pathways toward improved clinical care, offering a timely and integrated perspective on preeclampsia.

## The heterogeneity challenge: recognizing preeclampsia subtypes

A major advancement in preeclampsia research is the recognition that it is not a single disease but a spectrum of clinical and molecular subtypes. Notably, early-onset preeclampsia (EOP), which develops before 34 weeks of gestation, differs significantly in its underlying pathophysiology from late-onset preeclampsia (LOP), which occurs after 34 weeks. Han et al. illustrated through integrated weighted gene co-expression network analysis that severe and early-onset preeclampsia shows substantial molecular alterations between these subtypes. They found that EOP is associated with pronounced molecular disruptions, including two key gene modules linked to gonadotropin secretion, lipid storage, and chronic inflammation. In contrast, LOP displayed more subtle changes in placental gene expression, primarily involving stress-response pathways. In contrast, late-onset preeclampsia exhibits more nuanced changes in placental gene expression patterns. These distinct molecular signatures unique to each subtype carries significant implications for clinical management. EOP is marked by notable placental dysfunction with irregular trophoblast invasion and poor remodeling of spiral arteries, while LOP more often associated with maternal constitutional factors and cardiovascular risk. Comprehending these unique pathways allows for individualized therapeutic strategies. Importantly, Han et al. pointed out that abnormal placental lipid storage could be a contributing factor to the severity and EOP, emphasizing metabolic dysregulation as a key aspect shared among preeclampsia subtypes.

## Metabolic reprogramming: the cellular foundation of disease

Energy metabolism dysregulation has emerged as a central theme in preeclampsia pathogenesis, with mitochondrial dysfunction serving as a critical upstream mediator of disease progression. Li et al. identified multiple energy metabolism-related genes (MMRDEGs) through comprehensive bioinformatics analysis and several energy metabolism-related genes involvement in glycolysis, gluconeogenesis, lipid transport, and glucagon secretion. Among the most consistently dysregulated genes, including CRH (Corticotropin-Releasing Hormone), LEP (Leptin), PDK4 (Pyruvate Dehydrogenase Kinase Isozyme 4), SPP1 (Secreted Phosphoprotein 1), and SST (Somatostatin) demonstrated consistent dysregulation across preeclampsia cohorts, with qRT-PCR validation confirming increased LEP and CRH expression alongside altered SPP1 levels in preeclampsia samples.

Importantly, the identification of mitochondrial energy metabolism-related differentially expressed genes has provided new insights into disease mechanisms. Li et al. established that genes including OCRL, TPI1, GAPDH, and LDHA form diagnostic models with promising predictive performance, predominantly enriched in pyruvate metabolism, glycolysis, and ATP metabolism pathways. This metabolic dysfunction appears to drive oxidative stress and inflammatory responses through immune modulation, with CIBERSORT analysis highlighting significant variations in immune cell composition between preeclampsia and control groups, creating a pathological cascade that culminates in the clinical syndrome of preeclampsia.

## Inflammatory networks and immune dysfunction

The inflammatory aspect of preeclampsia encompasses much more than mere maternal immune activation, involving intricate networks of cellular stress responses and immune regulation disorders. Zhao et al. discovered that BNIP3-driven mitophagy is a novel mechanism linking cellular stress intensifies placental damage through activation of the NLRP1 inflammasome. Their study showed that both BNIP3-mediated mitophagy and NLRP1 inflammasome activation occur in mouse models of L-NAME-induced preeclampsia and in human placentas affected by preeclampsia. Notably, knockdown of BNIP3 in JEG3 cells preventing mitophagy and NLRP1 inflammasome activation upon subjected to hypoxia and reoxygenation. This pathway highlights the direct connection between mitochondrial dysfunction and inflammatory responses, with mitochondrial reactive oxygen species (mtROS) acting as a crucial mediator. Zhao et al. illustrated that silencing BNIP3 results in a notable decrease in mitochondrial damage and mtROS production., Furthermore, treatment with the antioxidant MitoTEMPO after BNIP3 silencing led to an even greater reduction in NLRP1 expression. Crucially, BNIP3 knockdown mitigates placental damage in preeclampsia mouse models, establishing a definitive therapeutic target for potential intervention.

### Cell-free DNA: a window into placental pathology

Cell-free DNA (cfDNA) analysis represents one of the most clinically promising advancements in preeclampsia research, offering non-invasive insights into placental health and disease progression. In a comprehensive review, Guo et al. outlined the multidimensional roles of cfDNA in preeclampsia. Their review highlighting quantitative alterations in cfDNA, fragmentomic profiles, and placenta-specific methylation patterns such as RASSF1A that demonstrate significant value for early prediction and severity stratification of PE. The mechanistic basis of cfDNA release involves placental hypoxia-induced trophoblast apoptosis, epigenetic dysregulation activating TLR9/NF-κB inflammatory pathways, and oxidative stress-mediated mitochondrial cfDNA fragmentation.

Guo et al. emphasized that integrating cfDNA with complementary biomarkers enhances predictive performance beyond what can be achieved with traditional clinical parameters alone. However, they acknowledged that challenges remain regarding preanalytical variability and dynamic gestational changes, with assay standardization constituting the fundamental translational bottleneck. Their review advocates for advancing fragmentomics-integrated multi-omics frameworks for precision prediction, representing a critical step toward personalized preeclampsia management.

## Therapeutic horizons: from bench to bedside

Secondary prevention methods for preeclampsia are progressing beyond the conventional approaches of low-dose aspirin and calcium supplementation. Akbar et al. offered an extensive review of pharmacological strategies for secondary prevention, showing that low-dose aspirin (LDA) can effectively reduce the incidence of early-onset preeclampsia (EOP) when initiated before 16 weeks of gestation. They noted that calcium supplementation benefits women who have inadequate dietary calcium intake. Concurrently, low molecular weight heparins (LMWH) appear to be promising, albeit with limited use for patients with a history of severe placental vasculopathy. New treatment targets are being explored, including metabolic modulators like metformin, which Akbar et al. suggested may reduce preeclampsia rates due to its anti-inflammatory and vascular benefits, especially in women with significant obesity. Statins, such as pravastatin, have demonstrated positive outcomes in lowering the incidence of preterm preeclampsia and enhancing maternal-fetal health through their multifaceted cardiovascular protective properties. Recognizing oxidative stress as a key pathogenic factor has prompted investigations into targeted antioxidant therapies. Afrose et al. assessed treatments aimed at oxidative stress in *in vitro* models of placental stress, revealing that agents like AD-01 and resveratrol may have therapeutic value by mitigating oxidative stress-related cellular dysfunction. Their research indicated that metformin could alleviate increases in uric acid and malondialdehyde induced by DMOG, Rho-6G, or TNF-α, while AD-01 effectively reduced both markers under various stress situations. These results bolster the advancement of precision medicine strategies aimed at addressing specific molecular pathways based on individual risk factors and disease classifications.

## Clinical context: comprehensive outcome assessment

The clinical implications of these molecular discoveries must be understood within the broader context of maternal and fetal outcomes. Ibeh et al. provided valuable insights through their retrospective analysis of 151 pregnant patients with thrombocytopenia, demonstrating that hypertensive disorders in pregnancy (including preeclampsia) are associated with higher neonatal intensive care unit transfer rates and lower birth weights in newborns. Their study revealed that thromboelastography (TEG) parameters correlate with pre-delivery platelet count in moderate and severe thrombocytopenia groups, suggesting that patients with preeclampsia-associated thrombocytopenia may have significant changes in blood coagulation and fibrinolysis systems requiring enhanced monitoring.

## Mitochondrial biomarkers in preeclampsia

The identification of mitochondrial and programmed cell death (mtPCD) biomarkers represents a significant advancement in understanding preeclampsia pathogenesis., Moving beyond traditional clinical parameters, these findings reveal fundamental cellular mechanisms driving this devastating pregnancy disorder. Through sophisticated bioinformatics integration of multiple datasets and rigorous experimental validation, Lin et al. have identified four critical genes—*SLC25A5*, *ACSF2*, *MFF*, and *PMAIP1*—that offer both diagnostic precision and mechanistic insights into preeclampsia development, suggesting a coordinated disruption of mitochondrial homeostasis and cellular survival pathways that fundamentally alter placental function. Particularly compelling is the identification of regulatory networks, including the *KCNQ1OT1*/hsa-miR-200b-3p/*ACSF2* axis, which opens new therapeutic intervention strategies. Additionally, drug predictions analysis identified clodronic acid offer immediate translational potential. This mtPCD-focused approach not only enhances our diagnostic capabilities through machine learning-validated biomarker panels but also fundamentally reframes preeclampsia as a disorder of mitochondrial dysfunction and programmed cell death dysregulation, positioning mitochondrial biology as a central therapeutic target for improving maternal and fetal outcomes in this complex pregnancy syndrome.

## MicroRNA biomarkers herald a new Era in pregnancy risk prediction

The integration of molecular biomarkers into routine prenatal care represents a paradigm shift toward precision obstetrics, with a microRNA (miRNA) profiling emerging as a potential tool for this transformation. A comprehensive retrospective study using over 600 pregnancies, Hromadnikova et al. has demonstrated that combinations of cardiovascular disease-associated miRNAs with maternal clinical characteristics can achieve remarkable predictive accuracy for adverse pregnancy outcomes. Detection rates exceeding 80% for most complications including preeclampsia (83.33%), HELLP syndrome (92.86%), and gestational diabetes requiring therapy (89.47%). Particularly striking is the 91.67% detection rate for stillbirth using miRNA biomarkers alone, obtained from a simple blood draw between 10–13 weeks of gestation. This approach transcends traditional risk assessment by identifying molecular signatures of cardiovascular dysfunction that underlie many pregnancy complications, enabling early intervention strategies that could fundamentally alter pregnancy trajectories. The cost-effectiveness and accessibility of this miRNA-based approach make it particularly promising for widespread implementation, potentially transforming pregnancy care from reactive management to proactive prevention across diverse healthcare settings.

## Challenges and future directions

Despite remarkable progress in understanding preeclampsia’s molecular foundations, significant translational challenges persist. The complexity of preeclampsia’s pathophysiology, with multiple interacting pathways involving metabolism, immunity, and vascular function, require a systems-level approaches rather than single-target interventions are unlikely to address its multifactorial nature. As emphasized by Guo et al. standardization of analytical platforms, particularly for cfDNA fragmentomics and multi-omics integration, represents a fundamental bottleneck for clinical translation. Additional limitations include the small and non-diverse cohorts in most studies, the lack of longitudinal validation, and practical feasibility Research Topic such as high costs and variability across healthcare systems.

Future research priorities should include the validation of molecular biomarkers in diverse populations, the development of point-of-care diagnostic platforms, and clinical trials of combination therapeutic strategies targeting multiple pathogenic pathways simultaneously. The integration of artificial intelligence (AI) and machine learning approaches with multi-omics data, as demonstrated by Wang et al. offers unprecedented opportunities for developing personalized risk prediction models and treatment algorithms.

## Conclusion

The merging of genomics, metabolomics, and systems biology is revolutionizing our comprehension of preeclampsia, changing it from a poorly understood syndrome to a Research Topic of related disorders with specific molecular pathways that can be targeted. Studies such as identifying subtype-specific signatures (Han et al.), illustrating metabolic reprogramming patterns (Li et al.), mapping inflammatory networks (Zhao et al.), and highlighting cfDNA biomarkers (Guo et al.), offers a thorough guide for precision medicine approaches to this complex Research Topic. Future efforts must also focus on addressing global disparities, ensuring that biomarker-based diagnostics and preventive strategies are accessible in low-resource settings, while also recognizing the long-term cardiovascular risks for mothers and developmental consequences for children. As we move closer to applying these findings clinically, our attention must transition from simple descriptive molecular profiling to validating functionality and targeting therapeutics, as demonstrated in the therapeutic studies conducted by Afrose et al. and the extensive prevention strategies proposed by Akbar et al. Mitochondria-based biomarkers by Lin et al. provided a clue for early detection of PE as well as miRNA-based biomarkers by Hromadnikova et al. The primary objective remains evident: to convert preeclampsia, currently a major cause of maternal and perinatal mortality, into a condition that can be prevented and managed through early prediction, targeted prevention, and personalized treatment plans. The way ahead necessitates ongoing interdisciplinary collaboration among basic scientists, clinicians, and translational researchers, along with a sustained commitment to standardization initiatives and clinical validation research. Only through such coordinated efforts can we fully achieve the potential of precision medicine to enhance outcomes for preeclampsia and related complications during pregnancy.

